# Building leadership skills through applied learning in an MPH program

**DOI:** 10.3389/fpubh.2025.1610306

**Published:** 2025-06-11

**Authors:** Sophie G. Wenzel, Pelagie Ky, Nicole Holt, Erika L. Austin

**Affiliations:** Department of Population Health Sciences, Virginia Tech, Blacksburg, VA, United States

**Keywords:** leadership, communication, negotiation, interprofessionalism, public health

## Abstract

Strong leadership capacity, including both knowledge of leadership theories and having the requisite leadership skills, is essential for effective public health practice in an increasingly complex and politically challenging environment. Master of Public Health programs are well-positioned to provide this leadership training to future leaders. Using high impact educational practices and principles of adult learning, Virginia Tech’s Public Health Program created a master’s level Public Health Leadership and Interprofessionalism class. Topics covered include leadership, interprofessionalism, negotiation, ethics and professionalism, adaptive leadership, systems thinking, models of team effectiveness, communication, change management, workplace diversity, strategic planning and personal leadership. All students completed a baseline self-assessment for all program competencies upon program matriculation and again at the time of their graduation. We found a statistically significant increase in all competencies covered in the leadership class. In addition, we identified five overall topics that students said were crucial in their public health practice: understanding strengths-based leadership, focusing on dynamics of team effectiveness and team roles, understanding the role of communication as a leadership tool, and applying negotiation and interprofessionalism skills. Overall, we have evidence that the class was effective and provided students with concrete skills that they can refer back to once they enter the public health workforce.

## Introduction

Strong leadership capacity, including both knowledge of leadership theories and having the requisite leadership skills, is essential for effective public health practice in an increasingly complex and politically challenging environment. Across sectors, however, the development of leaders with both concrete leadership skills and knowledge of the science underlying public health practice continues to be a challenge. Indeed, the 2021 Public Health Workforce Interests and Needs Survey (PH WINS) noted leadership training as a top self-identified need among public health workers across sectors and job types (1, 2). In addition, the majority of individuals in public health practice do not have formal training in either the science of public health nor in public health leadership; this is especially true among individuals working in state and local health departments, with estimates suggesting that fewer than 1 in 5 workers holds a degree in public health (1, 3). The large number of public health leaders reaching retirement age, coupled with the exodus of public health leaders following the Covid-19 pandemic, has increased the need for targeted leadership training for the next generation of public health leaders (4). Public health education, and the Master of Public Health degree in particular, is well-positioned to provide much-needed leadership training.

## Background

Public health education has shifted over the past decade to focus more explicitly on developing the skills graduates will need to be effective public health practitioners. The Council on Education for Public Health (CEPH), the accrediting body for public health degrees at the Bachelor’s, master’s, and doctoral level, has been a leader in this change. While the 2011 CEPH accreditation criteria focused on core knowledge in the major fields of public health (e.g., biostatistics, epidemiology, social and behavioral science, environmental health science, and health administration), 2016 saw a significant overhaul of the CEPH accreditation criteria with the introduction of 22 skills-based competencies required of Master of Public Health graduates (5, 6). These competencies were developed through an extensive external review process that sought to prioritize the skills most critical to public health practice, including several competencies focused on leadership (7). Likewise, the Certified in Public Health exam, a credential available to graduates and individuals with public health workforce experience, features leadership as one of ten domains in its revised 2024 content outline (8) (developed based on a job task analysis of public health practitioners completed in 2022). Similarly, the Council on Linkages between Academia and Public Health Practice Core Competencies also features leadership as one of eight domains in its detailed listing of the skills necessary for effective public health practice (9).

The Covid-19 response highlighted challenges in workforce development and preparedness, especially in leadership, on a national and global level. The public health workforce operated under unprecedented strain and leaders left the workforce at alarming rates due to increasing pressures. In response, the Association of Schools & Programs of Public Health (ASPPH) created a national leadership training agenda to assess and define essential leadership competencies for the current and future public health workforce (10). With public health professionals coming from various backgrounds and experiences, ASPPH argues that incorporating these skills and competencies into public health curricula prepares students and professionals to be successful in future leadership roles (10).

The shift toward skills-based public health education has necessitated a rethinking of how such education is delivered; this is particularly true at the master’s level, where students are primarily adult learners, some of whom already have work experience. Adult learning theory is a useful framework for supporting this shift as it explicitly recognizes the different perspective from which adults approach learning given their life experiences. Particularly in settings where some students and/or the instructor have public health practice backgrounds to draw on, the critical need for leadership skills in public health practice can be motivated by discussions of their experiences with both effective and ineffective leaders, thus providing the “why” that adult learners expect when learning new material. High impact educational practices, many of which feature real-world examples and authentic application of learning are also well-suited to the development of leadership competency among master’s-level students (11). Experiential learning approaches, which emphasize thoughtful reflection on new material prior to attempting its application, are ideal for developing competency in practice-based leadership skills for adult learners (12, 13). Many MPH students have little actual work experience, thus the experiential learning approach gives them the necessary frame of reference to apply material learned in the classroom.

## Pedagogical framework

To address the need for public health graduates with strong leadership skills, a 2-credit, required Public Health Leadership and Interprofessionalism class, taught from an adult learning perspective, was created at Virginia Tech. The Leadership class covers CEPH competencies 16 (Apply leadership and/or management principles to address a relevant issue), 17 (Apply negotiation and mediation skills to address organizational or community challenges) and 21 (Integrate perspectives from other sectors and/or professions to promote and advance population health). Other topics covered in the class include ethics and professionalism, adaptive leadership, systems thinking, models of team effectiveness, communication, change management, workplace diversity, strategic planning and personal leadership.

In 2015, Lachance and Oxendine (14) authored a paper calling for public health programs to prioritize the teaching of technical and leadership skills equally due to the acute need for leaders to replace the aging workforce. Lachance and Oxendine (14) state that technical skills alone are not enough to effectively influence population health, and argue that students should develop complementary leadership skills.

In addition, cultivating an understanding of conflict resolution and negotiation allows for students and future professionals to manage difficult conversations with empathy in order to reach mutually beneficial outcomes (15). Finally, interprofessionalism is a core public health leadership skill, as professionals often work with others in fields adjacent to public health. In an academic setting, interpersonal education allows students to practice teamwork and collaboration skills, and when students have the opportunity to collaborate across disciplines or concentrations, it better prepares them to employ these skills in a real life setting (16).

In order to create the class, the instructor conducted a systematic content analysis of masters level public health leadership classes and other classes that incorporated relevant competencies in other accredited schools and programs. The instructor reviewed textbooks and literature on the topic to determine the most appropriate and relevant class content. Material was also adapted from CEPH-provided assessment examples.

The instructor used high impact educational practices to develop the class to ensure students had a meaningful learning experience (11). Students participate in collaborative assignments such as case studies and group negotiations. Students apply their learning to real-world examples, including solving real case studies and participating in negotiations on relevant topics such as vaccination policies and school based sexual health education. Students have the opportunity to interact with each other during class, provide and receive feedback regularly, and participate in active reflection on their learning.

The class uses the principles of experiential learning (13) to motivate students to learn. Typically, the first half of class is lecture based with plenty of opportunities for small group discussion, such as think-pair share, and reflection. The second half of class is usually an activity to practice what was learned in lecture. An example of this is a hands-on, scenario-based message mapping exercise and mock press conference after a class on risk communication. Each competency is assessed as described in Table 1.

**Table 1:** Class assessment by competency.

Competency	Class assessments
16-leadership	Students participate in team-based public health leadership case studies, present their results orally, and write a reflection paper
17-negotiation	Students participate in a team-based role playing exercise in which they have to negotiate a vaccine or sexual health education policy for a school board. Students write a paper on how they incorporated principles of negotiation in their discussion
21-interprofessionalism	Students pair up with someone outside the public health discipline, either in practice or in academia (ie: nursing, engineering, geography, water and sanitation, etc.) to engage in a conversation about how they would solve one of the United Nations’ sustainable development goals. Students must spend at least an hour conversing, and each person must share their approach to solving the problem identified from their discipline’s perspective. Students then write a paper about the process and specify how they applied each of the Interprofessional Education Collaborative (IPEC) competencies during their conversation

The instructor consulted frameworks for best practices in adult leadership to develop the course and incorporated these principles into the instructional pedagogy. Principles of adult learning that were incorporated into the class include: (1) Adults need to know why they need to learn something (2) Adults need to build on their experience, (3) Adults have a need to feel responsible for their learning, (4) Adults are ready to learn if training solves an immediate problem (5) Adults want their training to be problem focused, (6) Adults learn best when motivation comes intrinsically (12, 17).

The ultimate goal of the class is to teach new relevant skills and give students the opportunity to apply those skills within the classroom setting. Students leave the class with new skills, tools and strategies to serve as public health leaders.

## Learning environment

The two-credit class, taught in person, is required for all first year MPH students regardless of concentration. It is part of the shared core of courses that includes policy and administration, behavioral health, program planning and evaluation, environmental health and epidemiology and quantitative methods. The class has been taught since 2019 and average class size is 38 (range: 26–48.) Class is taught by one faculty member with a DrPH in public health leadership who has years of practice experience, and includes two guest speakers on special topics who are experts in their field (ethics, diversity in the workplace.)

Learning outcomes for the class include:

- Apply requisite management and leadership skills for public health practice including effective decision-making, shared vision, collaboration, communication and ethics
- Demonstrate mediation and conflict resolution skills
- Describe core concepts of negotiation, including finding common interests, values, communication styles and shared agendas
- Describe core concepts of team building in public health settings and provide reflective introspection on the use of these concepts
- Develop awareness and appreciation of the roles and contributions of various public health, healthcare professionals and other partners in protecting the public’s health through interprofessional teams

## Results

Our first assessment question concerned the effectiveness of the course, specifically: Did students’ self-assessed competency on the three foundational competencies covered in the class (CEPH 16, 17, 21) increase significantly from baseline (before enrolling in the course) to follow-up (after completing the course)? All students completed a baseline self-assessment for all program competencies upon program matriculation and again at the time of their graduation. Students reported their perceived competency level on a scale of 1 to 5 with 1 being Fundamental Awareness, 2 being Novice, 3 being Intermediate, 4 being Advanced, and 5 being Expert. We found a statistically significant increase in all competencies covered in the leadership class (Table 2). Students’ average rating of their competency in leadership (16) increased from program entry (*M* = 2.44, SD = 0.923) to graduation (*M* = 3.76, SD = 0.783); t (187) = 1.97, *p* < 0.001. Competency in negotiation (17) also increased from program entry (*M* = 2.26, SD = 0.926) to graduation (*M* = 3.68, SD = 0.897); t (170) = 1.97, *p* = <0.001. Competency in interprofessionalism (21) saw the largest average gain from program entry (*M* = 2.53, SD = 0.979) to graduation (*M* = 4.18, SD = 0.734); t (197) = 1.97, *p* < 0.001.

**Table 2:** Means for baseline and graduation competency assessment (2020–2023).

	FC 16 *n* (mean)	FC 17 *n* (mean)	FC 21 *n* (mean)
Baseline	140 (2.44)	137 (2.263)	159 (2.54)
Graduation	80 (3.76)	80 (3.68)	78 (4.18)

We also asked the research question: “What did students perceive as most helpful about the course, in light of their experience in public health practice?” To answer this question, key informant interviews were conducted with four students who had either been in practice before taking the class, were in practice while they took the class, or have graduated and held a practice position since taking the class. Students identified five overall topics that they said were crucial in their public health practice.

- All of the students mentioned the importance of understanding strengths-based leadership. One student said: “We always need to practice working with people who see things from different perspectives. You can identify everyone’s strengths and create a plan as a team that leverages everyone’s strengths.”
- Students also appreciated the class’s focus on understanding the dynamics of team effectiveness and team roles. As one student illustrated: “Thinking about team work is integrated across class. It forces you to be reflective about how you are showing up.”
- Students mentioned the class’s emphasis on communication. The message mapping developed during class was helpful as some of the students navigated vaccine hesitancy discussions as Covid-19 community health workers. Learning how to communicate across communication styles also proved helpful as they communicated with co-workers and supervisors.
- Having the opportunity to practice a negotiation gave students skills that they could use in practice. Students said those skills have helped them navigate complicated situations in the community and workplace. One student shared that she learned that “conflict does not need to be negative, it can be productive. Addressing it directly is good.”
- Finally, students appreciated the opportunity to learn about and practice interprofessionalism. As one student noted: “The [interprofessional activity] was super cool. It was an example of interprofessionalism-everyone had a role. It shows in reality this can really happen. It was a good taste of collaboration and why it’s important to have a diverse team.”

Two of the students reflected on the class and its benefits after having been in practice for several years. As one student said: “You hear about it in a class and it’s an idea, and then you get to the community and experience it, you can identify the bigger ideas you talked about… interprofessionalism, leadership styles. Seeing it in action is really helpful. It goes from an idea to real life. ‘Oh so that’s why you learn about that!’ It feels more tangible.”

Another student concurred with this idea: “I do not think I realized how important these things would be for my day to day. I had not had experience in public health. I did not take as much advantage of the class as I should have. Now looking at all the themes, these topics would be so helpful.” She proceeded to say that if the instructor were to offer a refresher workshop now, she’d like to take it.

When asked what changes they perceived in themselves after taking the class, students talked about a gain in self-awareness, a desire to be more intentional in their role, and finding more initiative when it came to communicating with peers and superiors.

## Discussion

Leadership skills are an important component of public health training. We used several data sources to assess a master’s-level leadership course designed using high-impact educational activities, consistent with adult learning theory. We noted that students’ self-assessed competency on all three foundational competencies related to leadership increased significantly from baseline (at the start of their master’s program) to follow-up (at the time of their graduation from the program). Qualitative feedback suggested that students appreciated linking knowledge gained through the course (e.g., leadership styles, roles on teams) with experiential learning activities, which is a strong example of the integration characteristic of high-impact educational practices. Notably, students also recognized how other “power skills” – particularly communication – interact with and enhance leadership competency. Overall, we have evidence that the educational intervention was effective and provided students with concrete skills that they referred back to once they entered the public health workforce.

Today’s public health graduates will become tomorrow’s public health leaders, necessitating innovative and evidence-based emotional intelligence, and critical thinking skills. Educators should respond to graduates’ desire for ongoing leadership training, extending the same active learning approaches for workforce development training. Such trainings could not only reinforce public health graduates’ previous learning, but could also support the leadership potential of the vast number of public health workers without formal public health training. Future trainings should further integrate factual information on leadership theories and concepts with an even greater emphasis on related “power skills” such as communication, team-work, emotional intelligence, and interpersonal skills.

## Data Availability

The raw data supporting the conclusions of this article will be made available by the authors, without undue reservation.
